# Genetic alterations of JAK/STAT cascade and histone modification in extranodal NK/T-cell lymphoma nasal type

**DOI:** 10.18632/oncotarget.3776

**Published:** 2015-04-25

**Authors:** Seungbok Lee, Ha Young Park, So Young Kang, Seok Jin Kim, Jinha Hwang, Seungho Lee, Soo Heon Kwak, Kyong Soo Park, Hae Yong Yoo, Won Seog Kim, Jong-Il Kim, Young Hyeh Ko

**Affiliations:** ^1^ Genomic Medicine Institute (GMI), Medical Research Center, Seoul National University, Seoul, Korea; ^2^ Department of Biomedical Sciences, Seoul National University Graduate School, Seoul, Korea; ^3^ Department of Pathology, Samsung Medical Center, Sungkyunkwan University School of Medicine, Seoul, Korea; ^4^ Division of Hematology-Oncology, Internal Medicine, Samsung Medical Center, Sungkyunkwan University School of Medicine, Seoul, Korea; ^5^ Department of Internal Medicine, Seoul National University Hospital, Seoul, Korea; ^6^ Department of Molecular Medicine and Biopharmaceutical Sciences, Graduate School of Convergence Science and Technology and College of Medicine, Seoul National University, Seoul, Korea; ^7^ Department of Health Sciences and Technology, Samsung Advanced Institute for Health Sciences & Technology, Sungkyunkwan University School of Medicine, Seoul, Korea; ^8^ Department of Biochemistry and Molecular Biology, Seoul National University College of Medicine, Seoul, Korea

**Keywords:** extranodal NK/T-cell lymphoma nasal type, next-generation sequencing, JAK-STAT pathway, chromatin modification, somatic mutation

## Abstract

Extranodal NK/T-cell lymphoma nasal type (ENKL) is a rare type of non-Hodgkin lymphoma that more frequently occurs in East Asia and Latin America. Even though its molecular background has been discussed in the last few years, the current knowledge does not explain the disease pathogenesis in most cases of ENKL. Here, we performed multiple types of next-generation sequencing on 34 ENKL samples, including whole-exome sequencing (9 cancer tissues and 4 cancer cell lines), targeted sequencing (21 cancer tissues), and RNA sequencing (3 cancer tissues and 4 cancer cell lines). Mutations were found most frequently in 3 genes, *STAT3*, *BCOR*, and *MLL2* (which were present in 9, 7, and 6 cancer samples, respectively), whereas there were only 2 cases of *JAK3* mutation. In total, JAK/STAT pathway- and histone modification-related genes accounted for 55.9% and 38.2% of cancer samples, respectively, and their involvement in ENKL pathogenesis was also supported by gene expression analysis. In addition, we provided 177 genes upregulated only in cancer tissues, which appear to be linked with angiocentric and angiodestructive growth of ENKL. In this study, we propose several novel driver genes of ENKL, and show that these genes and their functional groups may be future therapeutic targets of this disease.

## INTRODUCTION

Extranodal natural killer (NK)/T-cell lymphoma nasal type (ENKL) is a rare histopathological subtype of lymphoma with unique clinical features and geographic variation. This lymphoma usually occurs in the upper airway tract, mostly in the nasal and paranasal area; however, around 20% of cases can occur in other tissues, including the skin, soft tissue, gastrointestinal tract, and testis [1–4]. Although most of the cases of ENKL are diagnosed in the early stage of the disease, patients usually have poor response to combination chemotherapy, resulting in 46%–60% long-term survival [3, 4]. Only half of the patients with advanced-stage disease can survive for 1 year, despite improvements in treatment [5, 6].

ENKL is strongly associated with Epstein–Barr virus (EBV) infection. EBV infection is thought to be an early event in the pathogenesis of the disease [7], and additional genetic alterations are essential to induce lymphomagenesis. Mutations in well-known tumor suppressor genes (TSGs), including *TP53* (20%–60% of cases) and *FAS* (around 50% of cases), have been reported [8], and array-based comparative genomic hybridization studies reported variable genetic changes, including gains in 1q21–q44, 2q, and 7q, and loss of 6q16–27 and 17p15–22. Among these lesions, the 6q region includes several TSGs, such as *PRDM1*, *FOXO3*, and *HACE1* [9, 10]. In particular, recent studies reported frequent *JAK3*-activating mutations in ENKL patients using next-generation sequencing (NGS), which suggests that the JAK/STAT signaling pathway is a key molecular factor in the pathogenesis of this disease [11, 12].

Previous gene expression profiling of NK cell malignancies led to the association between apoptosis, cell adhesion molecules/extracellular matrix (ECM) receptor interaction, and signal transduction pathways (including JAK/STAT, mTOR, etc.) with ENKL tissues [13]. MicroRNA dysregulation, which is significantly enriched among genes involved in cell cycle-related, p53, and MAPK signaling, was also suggested as a mechanism of lymphomagenesis [14]. However, the current knowledge on ENKL does not adequately explain the disease pathogenesis in most cases.

Although several studies have suggested a carcinogenic mechanism for ENKL, its molecular pathogenesis remains under-recognized. Because of the rarity of the disease and limitations in obtaining sufficient amounts of fresh tissues for molecular studies, to date, its study has been restricted to a few research groups. Here, we report genomic information on ENKL using the NGS method, and suggest several novel genes or pathways involved in the pathogenesis of this disease. In addition, we provide comparative data on gene expression profiles in primary ENKL tissues, NK-cell lymphoma cell lines, and normal NK cells, which support the genetic alterations discovered in the genomic sequencing experiments.

## RESULTS

### ENKL samples exhibited genomic heterogeneity and frequent mutations in TSGs

We performed WES on 9 ENKL tissue samples (cancer tissue, CT1–9) and 4 NK-cell lymphoma/leukemia cell line samples (cancer cell, CC1–4), with 4 paired normal blood (normal blood, NB1–4) and 3 unpaired NK cells from healthy volunteers (normal cell, NC1–3) as controls. A total of 251 somatic mutations, including 220 nonsynonymous single nucleotide variants (nsSNVs) and 31 coding insertions/deletions (indels), were identified from 4 paired samples. However, each mutation was identified in just 1 sample, and only 5 genes (*FGF10*, *KRAS*, *MLL2*, *FRY*, and *TP53*) were shared by ≥ 2 samples. These somatic variants were not detected further in 5 ENKL tissues and 4 NK-cell lymphoma cell line samples. Therefore, we extended our variant analysis to include RNA-Seq and targeted sequencing of 21 paraffin-embedded samples, which were analyzed together with 13 WES samples (Supplementary Tables 1 and 2).

**Figure 1:**
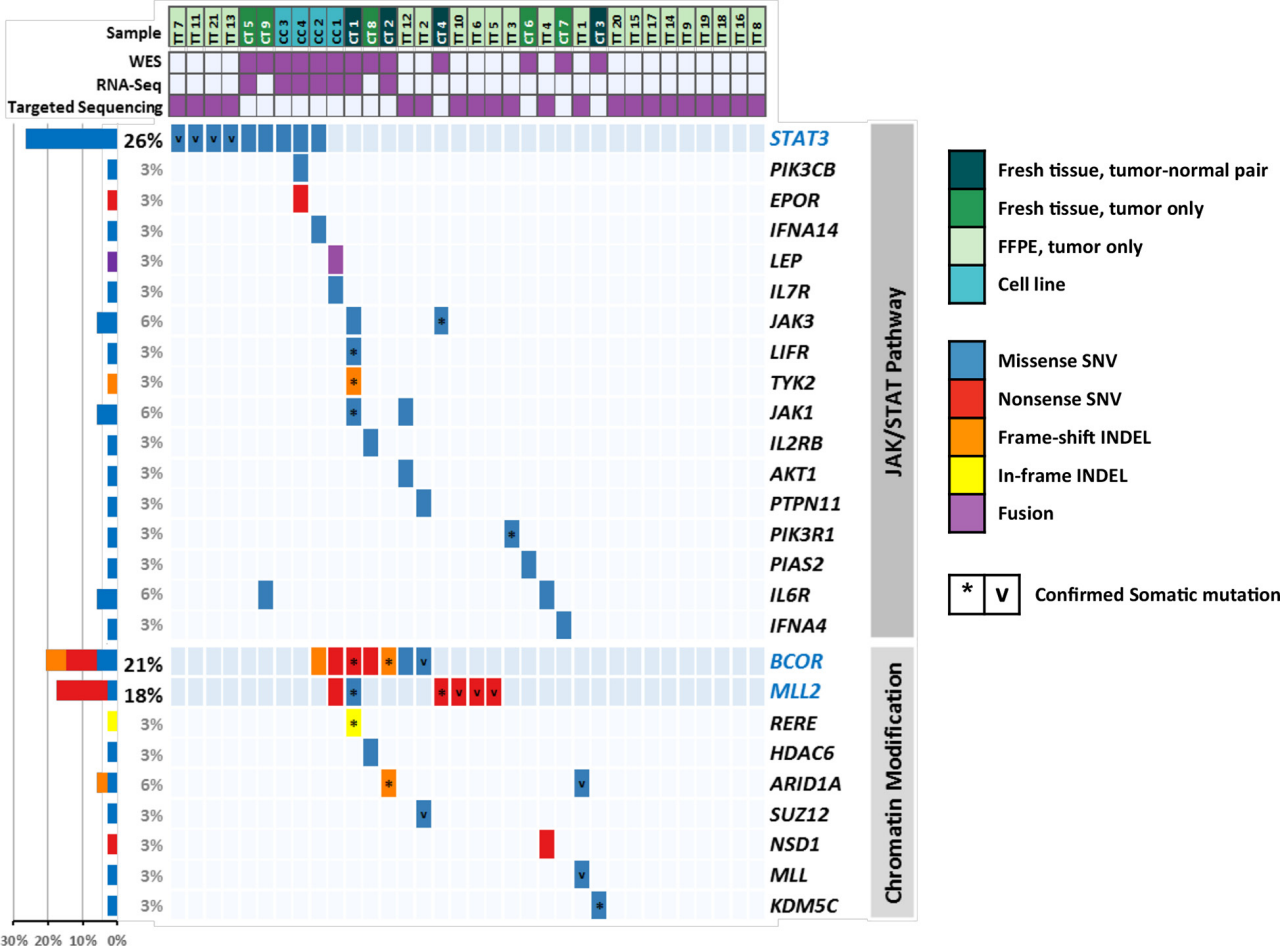
Distribution of mutations in ENKL

When all platforms were considered (WES, targeted sequencing, and RNA-Seq), *STAT3* was the most frequently mutated gene (9/34 cases, 26.5%), followed by *BCOR* (7/34 cases, 20.6%), and *MLL2* (6/34 cases, 17.6%) (Figure 1). Interestingly, their variants were nearly mutually exclusive of each other (*P* = 0.000044). Among the known ENKL-associated genes, mutations were most frequent in *TP53* (4/34 cases, 11.8%), followed by *KRAS* (2/34 cases, 5.9%), and *IL6R* (2/34 cases, 5.9%) [15]. In total, 22 cases had mutations in TSGs, including *MLL2*, *BCOR*, and *TP53*, 45% of which (10/22 cases) had at least 1 loss-of-function (LOF) mutation (nonsense SNV/frameshift indel), which implies that inactivation of TSGs may play an important role in ENKL pathogenesis. A full list of variants from study samples is shown in Supplementary Table 3.

Each column represents an ENKL case, which is shown in different colors according to the platform used. Each row represents a gene in the JAK/STAT pathway or chromatin modification gene ontology. Five mutation types are also distinguished by different colors. Asterisks (*) indicate somatic mutations in four tumor-normal pairs of exome sequencing. ‘v’ marks indicate mutations in targeted sequencing which confirmed to be somatic by Sanger sequencing of matched normal (uninvolved bone marrow) samples. Bars (left) represent the mutation rates in the 34 samples. ENKL, extranodal NK/T-cell lymphoma nasal-type.

### Genome analysis of ENKL revealed enrichment of alterations in the JAK/STAT pathway, among which *STAT3* was the most frequently mutated gene

**Figure 2:**
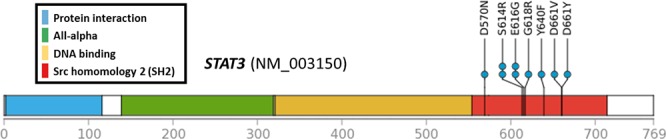
Locations of *STAT3* mutations All 9 missense SNVs were accumulated in the SH2 domain.

In JAK/STAT cascade genes, all mutations occurred at different positions, with the exception of 1 nsSNV of *STAT3* encoding p.Glu616Gly, which was identified in 2 out of 34 cases. Another 2 cases showed the same amino acid alteration (*STAT3* p.Ser614Arg), differing only at the nucleotide level. Interestingly, all mutations occurred at the SRC homology 2 (SH2) domain of STAT3 (Figure 2).

A recent study reported that activating mutations of *JAK3* (p.Ala572Val and p.Ala573Val) may play an important role in the pathogenesis of ENKL [11]. Because of the low coverage of WES in this region, we confirmed these mutations by Sanger sequencing. A total of 39 samples were used for checking *JAK3* hotspots, which included a subset of the samples used in exome and targeted sequencing and new 17 FFPE samples (ST1–17, Supplementary Table 4). Only 2 cases (2/39 cases, 5.1%) exhibited p.Ala527Val mutations, a frequency that was quite different from that mentioned in the report described above, which discovered either of 2 hotspot mutations in up to 35.4% of ENKL patients. We thoroughly examined the coding regions of *JAK3* by targeted deep sequencing again, but no additional mutation was found. However, genes included in the JAK/STAT signaling pathway [16] were mutated in about 55.9% (19/34) of all cases (Figure 1).

### Mutations in *MLL2* and *BCOR*, which are related to epigenetic regulation, were also frequent in ENKL

Mutations in *BCOR* were the second most common mutation (7/34, 20.6%), and about two-thirds of which were LOF mutations in exon 4 (1 frameshift deletion and 3 nonsense SNVs). Specific histone deacetylases (*HDAC1*, *HDAC3*, and *HDACB/5*) have been reported to interact with *BCOR* [17]. In addition, *MLL2* mutations occurred in 6 cases, and all except one were nonsense SNVs. *MLL2* is also known as *KMT2D* and encodes a lysine-specific methyltransferase that methylates the Lys-4 position of histone H3. Both *MLL2* and *BCOR* are classified into the same GO group, “chromatin modification” (GO:0016568) [18]; cases with mutated genes belonging to this category accounted for a total of 38.2% of cancers (13/34 cases) (Figure 1). Considering the mutual exclusivity between mutations in *MLL2* and *BCOR* and those of *STAT3* (Figure 1), epigenetic dysregulation might be another important feature of ENKL pathogenesis, together with the alterations of the JAK/STAT cascade.

### RNA-Seq revealed novel fusion genes and inactivation of *BCOR* as driver candidates

After the stepwise filtration of ambiguous calls from deFuse (described in the Methods), we selected 13 fusion candidates from cancer samples (Supplementary Table 5). These included *PDE4DIP* (*PARP8–PDE4DIP* in CC2), which was reported previously as fusion gene partners in hematologic malignancy [19]. We discovered an in-frame candidate among fusion genes, *SND1–LEP* of CC1, which was validated by Sanger sequencing. Even though its function in carcinogenesis remains unclear, the coverage depth pattern around breakpoints was distinctive from those of other samples. *LEP* is a member of the JAK/STAT signaling pathway and the fusion conserves most of the leptin domain. Supplementary Figure 1A shows *SND1-LEP* fusion, which were called again in TopHat-Fusion, and Supplementary Figure 1B and C shows the result of validation by RT-PCR and Sanger sequencing.

In addition to the fusion candidates mentioned above, we found BCOR inactivation in CC2, which was called in deFuse but predicted to be adjacent, alternative splicing, and deletion. CC2 is an NK-cell lymphoma cell line that was established from the circulating malignant cells of a 19-year-old female diagnosed with ENKL. The coverage pattern of WES also supported this mutation (Supplementary Figure 2); it was shown to be a homozygous deletion, although *BCOR* is located on X chromosome and this was a female sample.

We screened the expression levels of the TSGs that were selected from the cancer gene census, and found that 2 genes, *BCOR* and *SH2B3*, showed near-complete suppression (fragments per kilobase of transcript, per million fragments sequenced (FPKM) < 0.1), whereas every NC sample had FPKM > 1 (Supplementary Figure 3A and 3B). As previous studies reported that SH2B3 provides negative feedback on the JAK/STAT pathway [20], its expressional suppression might also affect the development of ENKL.

### The gene expression profiles of both ENKL tissues and cell line samples reflect JAK/STAT cascade dysregulation and epigenetic alteration

We conducted sample clustering analyses to compare the overall gene expression profiles of each gene, and found that samples were well clustered by their sample groups, CT, CC, and NC. However, CT was clustered together with NC, and not CC, even though they both represent ENKL cancer samples (Supplementary Figure 4A). One previous report listed differentially expressed genes (DEGs) of ENKL using array-based methods [13]. We compared their log_2_(fold change) values with those found here for CT and CC samples, respectively (Supplementary Figure 4B and 4C). Among DEGs that satisfied |log_2_(ratio)| ≥ 1 in both studies, 86.63% (175 out of 202) had the same direction in case of CT (*r*^2^ = 0.32), whereas only 68.84% (148 out of 215) had changes with the same direction in CC samples (*r*^2^ = 0.11); this pattern was concordant with the results of clustering, showing that CC samples were distant from CT. Upregulated genes tended to be well correlated with CT samples (CT, 150 out of 155; CC, 86 out of 148), whereas downregulated genes were correlated with CC samples (CT, 25 out of 47; CC, 62 out of 67).

**Figure 3:**
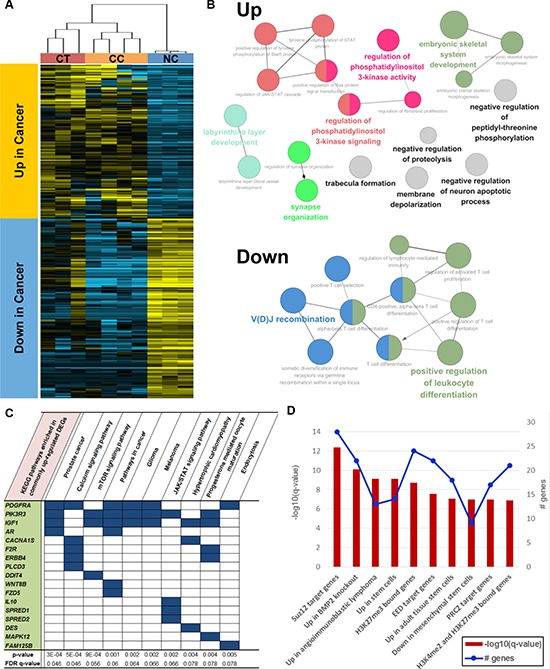
Functional enrichment of commonly upregulated or downregulated genes in both CT and CC samples **A.** Hierarchical clustering and heat map of common DEGs. **B.** Gene ontologies enriched in up- or downregulated genes. **C.** Top 10 KEGG pathways and **D.** top 10 CGP gene sets enriched in upregulated genes.

In the principal component analysis, CC was also separated from the other groups in the PC1 axis. However, in PC2, CT and CC were clustered together and were located apart from NC. Despite their huge difference, CT and CC samples shared 220 upregulated and 260 downregulated DEGs compared with NC samples (Supplementary Figure 4D and 4E, Figure 3A, and Supplementary Table 6). Upregulated DEGs were enriched in the GOs (biological process) regulating phosphatidylinositol 3-kinase (PI3K) activity, which include the JAK/STAT cascade, whereas downregulated DEGs were among the GOs (immune system process) related to T-cell immunity (Figure 3B, Supplementary Table 7). These results suggest that gene expression patterns also support major roles for the PI3K or JAK/STAT pathways in ENKL pathogenesis. Among the Kyoto Encyclopedia of Genes and Genomes (KEGG) gene sets, several pathways related to cancer were ranked in the top 10, in addition to “JAK/STAT signaling pathway” and “mTOR signaling pathway, ” which is located downstream of the JAK/STAT cascade (Figure 3C). We also found that several CGP gene sets related to the polycomb complex PRC2, which is involved in H3K27 trimethylation, were enriched among these upregulated genes (including the *Suz12* and *Eed* target genes). These findings support the contention that epigenetic dysregulation might be a key molecular factor in ENKL pathogenesis, as suggested in the mutation section (Figure 3D).

### Cancer tissue-specific DEGs are linked to the pathophysiologic features of ENKL

**Figure 4:**
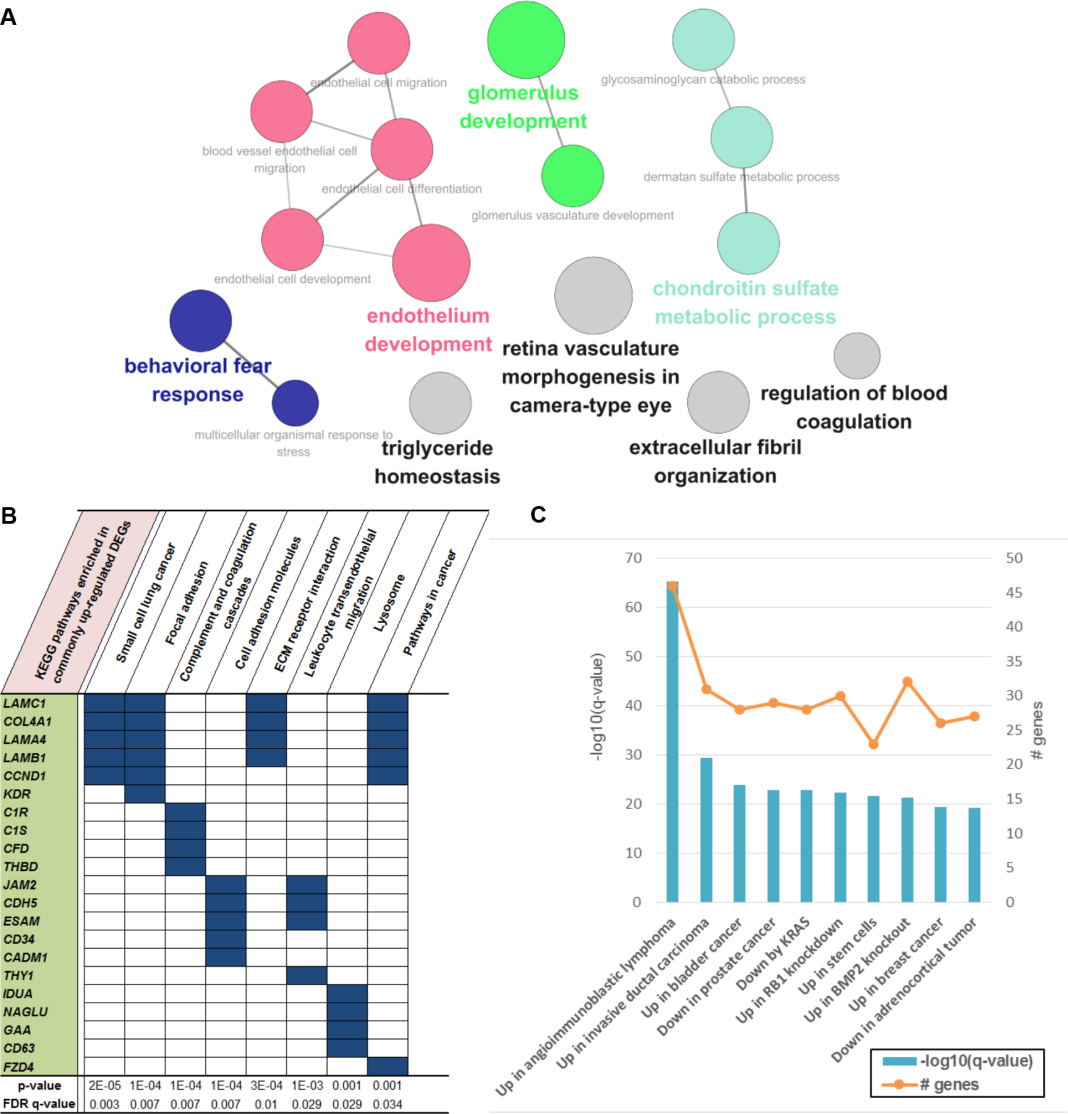
Functional enrichment of genes that were upregulated only in CT samples **A.** Gene ontologies enriched in CT-specific upregulated genes. There were several vasculature-development- or endothelium-development-related ontologies, which were the most significant. **B.** Top 10 KEGG pathways and **C.** top 10 CGP gene sets enriched in these genes.

As there was a huge difference between CT and CC regarding gene expression, we selected genes that were upregulated only in CT, which might reflect the tumor microenvironment of ENKL (Supplementary Table 6). As shown in Figure 4A and 4B, many gene sets involved in blood coagulation and vasculature development were significantly enriched in these DEGs, which was consistent with the results of a previous study that reported the activation of angiogenesis-related genes in ENKL tissues (Supplementary Table 7) [13]. Enriched pathways such as “focal adhesion” and “ECM receptor interaction” also suggest that these genes are activated during the interaction between cancer and the adjacent environment. In particular, gene sets such as “pathways in cancer” were also among the most significant gene sets, and might promote the development of ENKL. Interestingly, cancer-related CGP gene sets were even more significant in CT-specific DEGs than were those of commonly upregulated ones, such as genes that are activated in angioimmunoblastic lymphoma (Figures 3D and 4C).

## DISCUSSION

In this study, the molecular background of ENKL was explored using genome and transcriptome sequencing, which enabled us to discover novel drivers and therapeutic targets. We designed the study to screen exonic regions of known cancer genes, and mutations were shown to be enriched in *STAT3*, *BCOR*, and *MLL2*. In terms of functional gene groups, the JAK/STAT cascade and histones modification-related gene covered 55.9% and 38.2% of samples, respectively. Gene expression analysis also supported these functional groups in ENKL pathogenesis; moreover, we reported the cancer tissue-specific alterations at the molecular level.

Previous studies have suggested that the JAK/STAT and MAPK signaling pathways are crucial for disease development [13, 14]. In particular, frequent *JAK3*-activating mutations have shown that the JAK/STAT signaling pathway is a key molecular factor of ENKL [11]. However, the mutation frequency of *JAK3* (p.Ala572Val and p.Ala573Val) was much lower in our cases compared with previous reports (5.1% vs 35.4%). In a recent study of French population [12], *JAK3* mutations encoding p.Ala573Val were observed in the 15.8% of ENKL samples (3/19). In that study, most of the cases expressed phosphorylated JAK3 regardless of their *JAK3* mutation status (100% of mutant cases and 84.2% of wild type cases). Such result was also supported by a study of Japanese population that reported frequent *JAK3* phosphorylation and low *JAK3* mutation (5.0%). Another Japanese group even showed that no *JAK3* mutations (p.Ala572Val or p.Ala573Val) was found in a total of 49 samples which included 17 ENKL cases [21]. These results suggest that the function of *JAK3* might be altered mainly due to phosphorylation than mutation, and molecular backgrounds of JAK3-activation in ENKL might be different according to ethnicity. Instead of *JAK3* mutations, we identified several alterations in other components of the JAK/STAT pathway. About a quarter (26.5%) of our ENKL cases had nsSNVs in *STAT3*, which were clustered on the SH2 domain. Its pro-oncogenic role has been demonstrated by stabilizing via the formation of dimers with other STAT proteins through reciprocal SH2 domain interactions [22]. Frequent mutations in this domain can be presumed to be gain-of-oncogenic function mutations; however, additional studies are needed to confirm these mutations and their functional effects.

*STAT3* can be activated by extrinsic ways as well as by intrinsic ways. EBV, the well-known pathogen of ENKL, is one of the extrinsic sources of activation of *STAT3*; the latent membrane protein 1 of EBV was suggested as a STAT-activating agent in a mouse model [23]. Another study demonstrated that the Epstein–Barr nuclear antigen 2 also was a coactivator for the transcriptional enhancer of *STAT3* [24]. Our results can be partly interpreted as intrinsic components of *STAT3* activation. We found a novel fusion gene of *LEP* in 1 sample, the protein product of which (leptin) plays a role at the top of the JAK/STAT pathway as a stimulator of *STAT3* phosphorylation [25]. Loss of *SH2B3* has also been reported to increase *STAT3* phosphorylation [26], the expression of which was nearly completely suppressed in half of the cases (Supplementary Figure 3B). In addition to the inactivation of *SH2B3*, we found that *PIK3R3* was upregulated in all ENKL samples; this gene is also known as a downstream element of the JAK/STAT cascade and leads to cell proliferation and survival (KEGG pathways). Together with *STAT3* mutations, or independently, these alterations may play roles in ENKL pathogenesis.

Epigenetic dysregulation is an emerging part of cancer genomics, which is attributed to new genes discovered by NGS technology. The protein encoded by *MLL2* is a histone methyltransferase that targets the H3K4 site, and LOF mutation of *MLL2* has been reported in hematologic malignancy. In one recent study that was performed using NGS, B cell lymphomas had frequent somatic mutations in *MLL2*, most of which (83.3%) were LOF mutations, similar to ours (85.7%) [27]. Some types of solid tumors also had *MLL2* mutations, although those exhibited a somewhat lower proportion of LOF (11.8% of renal cell carcinoma [28] and 66.7% of medulloblastoma [29] cases). The effect of altered MLL2 might vary according to tumor type.

**Figure 5:**
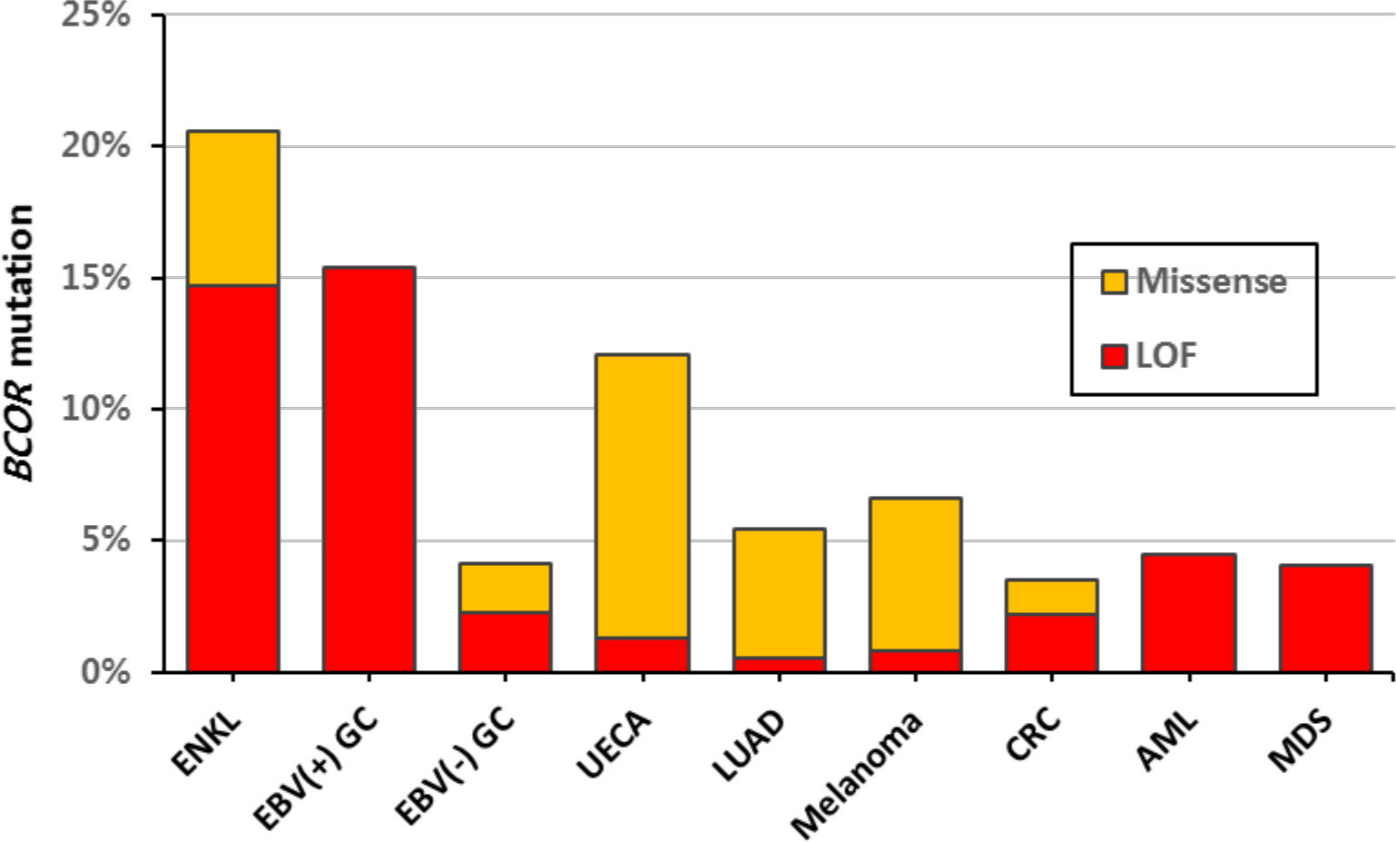
Mutation rates of *BCOR* according to tumor type EBV-associated malignancies (ENKL and EBV(+) GC) showed high percentages for both BCOR mutation rate and LOF proportion. LOF, loss-of-function; GC, gastric carcinoma; UECA, uterine endometrial carcinoma; LUAD, lung adenocarcinoma; CRC, colorectal adenocarcinoma; AML, acute myeloid leukemia; MDS, myelodysplastic syndrome.

*BCOR* was first reported as a corepressor of BCL-6 that is critical for germinal center formation [17], and its role in hematologic malignancy has been studied previously. Interestingly, most *BCOR* alterations occurred in male ENKL cases (6 mutations in men and 1 mutation in women), although this association was not statistically significant (27.3% of men vs 8.3% of women, *P* = 0.378). We tried to check whether mutations in *BCOR* in the X chromosome are related to the male predominance. However, *BCOR* mutation rates of male patients were lower in other male predominat malignancies (myelodysplastic syndrome (MDS), 3.8% of men vs 6.5% of women [31]; acute myeloid leukemia (AML), 5.5% of men vs 6.9% of women [32]; EBV-positive gastric cancer (EBV-GC), 14.2% of men vs 20% of women [33]). Next, we reviewed several public data to check whether BCOR mutations are associated with EBV infection-related malignancies. EBV-GCs, one of 4 molecular subtypes of gastric cancer, had frequent *BCOR* mutations (3 nonsense and 1 splicing) compared with the other 3 types (15.4% vs 4.8%, *P* = 0.051) [33]. With reference to the cBioPortal for Cancer Genomics (http://www.cbioportal.org), we selected several other solid tumors that are among the top 10 *BCOR*-mutated cancers and have available supplementary data sets (uterine endometrial carcinoma (UECA) [34], lung adenocarcinoma (LUAD) [35], melanoma [36], and colorectal adenocarcinoma (CRC) [37]), along with those of 2 hematologic malignancies, AML [32] and MDS [31]. Figure 5 shows the *BCOR* mutation rate and the proportion of LOF mutations for each cancer. EBV-associated malignancies (ENKL and EBV-GC) showed high percentages for both properties. UECA, which is not associated with EBV infection, exhibited a rather high *BCOR* mutation rate, but a low LOF proportion in *BCOR*, similar to LUAD and melanoma. The hematologic malignancies showed opposite patterns to those observed in UECA. In summary, EBV infection might be related to high mutation rates of *BCOR*, and LOF mutations seem to account for their majority.

As summarized above, *BCOR* mutation tended to occur significantly more frequently in EBV-associated malignancy (odds ratio = 3.74, *P* = 0.001) and mostly in the form of LOF. Previously, the oncogenic effect of EBV was explained by various epigenetic regulation mechanisms [38]. Considering that *BCOR* regulates chromatin modification by interacting with some histone-deacetylase-family genes, we were able to link the 2 major features, EBV infection and *BCOR* mutation, via an epigenetic mechanism. Overall, the mutations found in *MLL2* and *BCOR* led to the association between epigenetic dysregulation and ENKL pathogenesis. To the best of our knowledge, the present study was the first to identify mutations in chromatin-modifying genes in this disease. Together with the mutational features, RNA-Seq data also support this finding; upregulated genes were enriched in PRC2-related gene sets in gene expression analysis.

Until now, cell line samples have been used broadly in most types of cancer studies. For gene expression profiling, we designed a study to include both CT and CC samples, and tried to discover the common and different points between them. We found that CT and CC samples were quite different at the level of gene expression for both up and downregulated genes. The unsupervised clustering revealed even closer distance between CT and NC than that of CT and CC. As we selected study subjects which satisfied sufficient tumor cell contents, the difference could not be explained by normal cell contamination of tumor tissues. Due to the accumulated genetic alterations over division, the cancer cell lines may exhibit more prominent expression pattern far from primary tissue. Since we used the genes showing most variable expression patterns throughout samples for clustering analysis, these alterations specific for CC might affect greatly to the results. Although only some parts of DEGs were shared between the two groups, it seems meaningful that the DEGs included genes of JAK/STAT pathway or chromatin modification, supporting our main findings derived from mutational profiles.

Regarding genes that were upregulated only in CT, GO analysis showed that they were enriched in gene sets associated with angiogenesis, which is consistent with a previous study [13]. One possible explanation is that this might be a compensation for the vascular destruction caused by the angiocentric and angiodestructive growth of ENKL [39]. In addition, the pathways that are enriched among these genes include “focal adhesion” and “ECM receptor interaction, ” which include genes that mostly overlapped with those of “pathways in cancer.” As these types of pathways are associated with aggressive features of cancer [40], we suggest that the CT-specific changes might be linked to the characteristics of ENKL. These findings would have been missed if we analyzed cell line samples only, which do not include the effect of interaction between cancer and adjacent tissues, such as tumor microenvironment.

Here, we proposed several molecular candidates that can be applied as new therapeutic approaches using targeted agents. There seems the ethnic specificity of genetic backgrounds of ENKL, and more evaluations are required for these candidates in Asian populations. In addition to the validation of these candidates, it is necessary to subclassify ENKL patients according to their molecular backgrounds and check whether clinical features differ according to these groups, which will be feasible if a large sample size is used. We believe that the findings of this study will contribute substantially to the design of future studies of ENKL and of personalized approaches for cancer patients.

## METHODS

### NGS study subjects

In this study, we collected 34 cancer samples for sequencing, which included 9 fresh-frozen ENKL tissues (CT1–9), 4 NK-cell lymphoma cell lines (CC1–4), and 21 formalin-fixed paraffin-embedded (FFPE) ENKL tissues (TT1–21). The diagnosis of ENKL was established according to the 2008 World Health Organization classification. In all cases, the immunohistochemical study for CD3, CD56, TIA-1, and granzyme B, as well as EBER *in situ* hybridization was carried out using FFPE tissues. More than 70% of the infiltrated cells in the samples were positive for CD56. At least more than 70% of CD56-positive cells were positive for EBER *in situ* hybridization. In the CD56-negative case, the case was included for the study if EBV was positive in more than 50% of the infiltrating cells. For the frozen samples, frozen section was made for evaluation of cellularity and necrosis. As controls, 4 matched normal control samples (NB1-4, each paired with CT1-4) and NK cell samples from the peripheral blood of volunteers (NC1-3) were used [10]. For NGS, we extracted DNA from all study samples and RNA from some tissues (CT1, 2, and 5) and cell lines (CC1–4 and NC1–3). Supplementary Table 8 summarizes the information of the study subjects who were enrolled in this study. All patient samples were obtained in Samsung Medical Center, Seoul, Korea and this study was approved by the Institutional Review Board of the Samsung Medical Center, in accordance with the Declaration of Helsinki (approval number, 2013–12-076).

### DNA and RNA sequencing

DNA samples from 13 cancers (CT1–9 and CC1–4) and 7 normals (NB1–4 and NC1–3) underwent WES using SureSelect Human All Exon 50M (Agilent Inc., Palo Alto, CA). For the FFPE tissue samples, we applied targeted amplicon sequencing using the HaloPlex target enrichment kit (Agilent Inc.). The target regions were designed to include coding exons of ENKL-associated genes [15], cancer-driver genes [19], JAK/STAT signaling pathway genes, and BCOR network genes, together with those of genes with ≥ 2 somatic variants in 4 tumor-normal WES pairs. The full list of target genes is available in the Supplementary Table 9. Sequencing was performed on a HiSeq 2000 machine (Illumina, San Diego, CA), and the reads that were generated were aligned to the National Center for Biotechnology Information (NCBI) human reference genome (hg19) using Bowtie2 [41]. The mutated genes in GO group, “chromatin modification” (GO:0016568) and JAK/STAT signaling pathway of KEGG from targeted sequencing were confirmed to be somatic by Sanger sequencing on paired-normal DNAs, which were extracted from uninvolved bone marrow tissues. In all cases, bone marrow biopsy was performed as a staging work up. Bone marrow involvement was analyzed by FACS analysis for aspirate and EBER *in situ* hybridization for trephine biopsy. Mutual exclusivities of the genes were tested by Gitools [42]. We also checked for known *JAK3* mutations (p.Ala572Val, p.Ala573Val) by Sanger sequencing.

RNA was assessed for quality and was quantified using an RNA 6000 Nano LabChip on a 2100 Bioanalyzer (Agilent Inc.). The sequencing libraries were prepared as described previously and sequenced on a HiSeq 2000 machine (Illumina) [43]. The sequenced reads were aligned to the NCBI human reference genome (hg19) using the STAR 2-pass method [44, 45].

### Sequence variation analysis

In WES, reads marked as PCR duplicates were removed from downstream analysis (Picard, http://broadinstitute.github.io/picard/), and GATK was used to perform indel realignment and base quality score recalibration [46]. For somatic variant calling from 4 tumor/normal pairs, we used muTect and GATK SomaticIndelDetector to call SNVs and indels, respectively [47]. In the case of unpaired DNA samples, including those from FFPE tissues, GATK UnifiedGenotyper was used to call mutation candidates. All variants called were annotated in several genomic databases using ANNOVAR [48] and further narrowed down to driver candidates, as follows: (1) nonsilent SNVs or coding indels; (2) allele frequency = 0.0 in the 1000 Genomes Project, Exome Sequencing Project, and Complete Genomics sequencing data [49, 50]; (3) not shown in an additional 919 Korean exomes including NC1–3 (unpublished); and (4) located in genes other than those reported previously as having many false positives [51].

For variant calling from RNA sequencing data, we followed the best-practice recommendations of GATK, which include indel realignment, base recalibration, and a variant-calling process using HaplotypeCaller [46]. As a validation step, called variants were compared with those obtained in the genomic sequencing experiment described above.

### Fusion gene analysis

We used the deFuse tool to discover fusion transcripts in RNA-sequenced samples [52]. In addition to the default filtration, we tried to filter false-positive calls according to the following criteria: (1) every gene pair shown in NC samples was excluded; (2) adjacent gene fusions and those with gene distances < 200 kb were removed, unless they were predicted to be inversions or eversions; (3) fusions called as alternative splicing events were ignored; (4) fusions were supported by ≥ 5 spanning reads and ≥ 8 spanning mate pairs across breakpoints; (5) at least 1 gene in pairs was included in the RefSeq gene set; and (6) pairs of gene fusions were not paralogs of each other based on Ensembl version 72 [53, 54]. The fusions selected above were confirmed using the TopHat-Fusion caller with default options [55], and their genes were compared with the cancer gene census of COSMIC [56].

### Gene expression profiles of ENKL

We applied the HTSeq and DESeq2 tools to our RNA-Seq reads for gene expression analysis [57, 58]. Hierarchical clustering of samples was conducted with gene expression levels using Cluster 3.0, the results of which were visualized using Java Treeview [59, 60].

DEGs were defined as those with a *q*-value < 0.05 and |log_2_(fold change)| ≥ 1, thus differentiating 1 sample group from another. For further filtration, we also called DEGs for each cancer sample in comparison with NC. The genes selected here underwent gene set enrichment analysis (GSEA) [61]. Among the Molecular Signatures Database, which is a collection of gene sets for use with GSEA, we selected the following gene databases for analysis: curated gene sets including chemical and genetic perturbations (CGP) and KEGG gene sets. For gene ontology (GO) analysis, we used the ClueGO program, which is implemented in Cytoscape [62, 63].

## SUPPLEMENTAY FIGURES AND TABLES




