# Supplementary material for: Genetic alterations of JAK/STAT cascade and histone modification in extranodal NK/T-cell lymphoma nasal type
Source: Oncotarget. 2015 Apr 25;6(19):17764–76. doi: 10.18632/oncotarget.3776 (PMC4627344; doi:10.18632/oncotarget.3776)
Supplement: Supplementary file 1 [file oncotarget-06-17764-s001.pdf]

SUPPLEMENTAY FIGURES AND TABLES

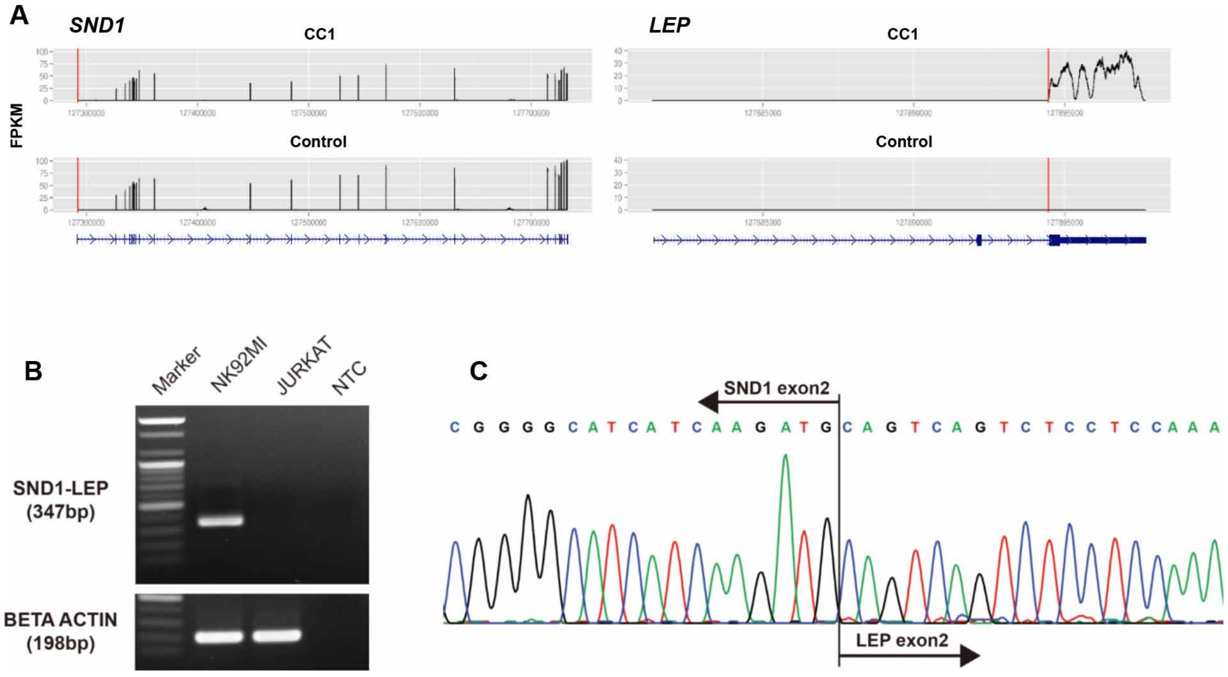

Supplementary Figure 1: In-frame fusion gene candidate in ENKL. A. Coverage pattern of the *SND1-LEP* fusion found in CC1, which was compared with the mean coverage of NC samples (Control). We assessed their expression levels in FPKM, and the predicted breakpoints are presented as red vertical lines. Their expression patterns around breakpoints were quite different from those of normal controls. FPKM, fragments per kilobase of transcript per million fragments mapped. **B.** RT-PCR detection of 347bp of *SND1-LEP* transcripts using primer set of *SND1 F* and *LEP R*. A specific band was amplified in NK92MI(CC1) cell line. JURKAT cell line is a negative control. NTC(No Template Control) is RT-PCR products, which lack temeplate. **C.** The sequencing analysis of the *SND1-LEP* revealed that *SND1* exon2 was fused in-frame with *LEP* exon2.

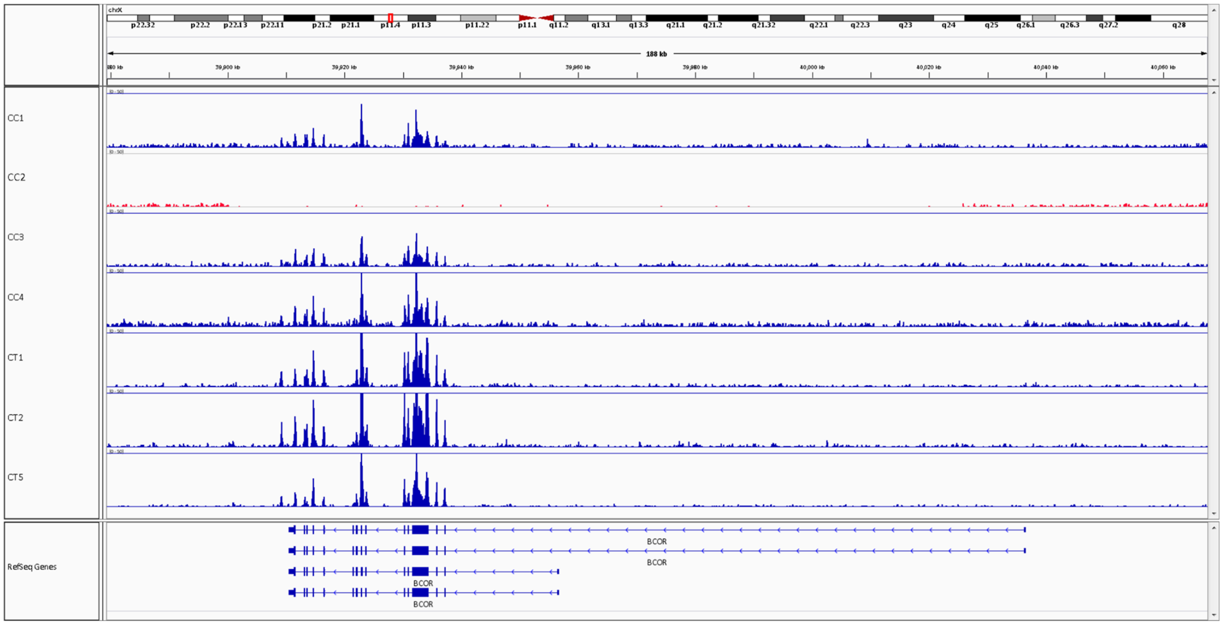

Supplementary Figure 2: Coverage patterns of *BCOR* in WES. This figure displays the read depth of *BCOR* region in WES samples. CC2 was predicted to have *BCOR* deletion in the RNA-Seq analysis, and now it shows the very low read depth throughout the region in WES. It suggests that CC2 has the homozygous deletion of *BCOR* region.

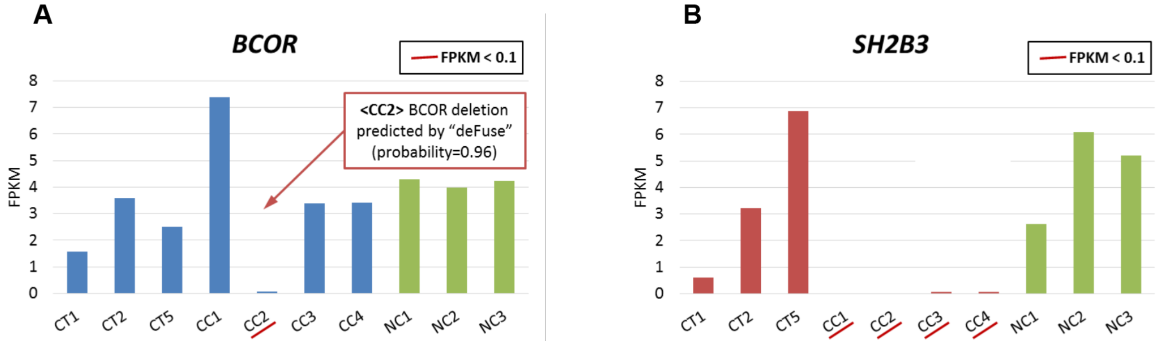

Supplementary Figure 3: Inactivation of TSGs in ENKL. A. Near-complete suppression of BCOR in CC2. The expression of BCOR was FPKM < 0.1 in CC2, and deFuse predicted the deletion of BCOR in this sample. **B.** Near-complete suppression of SH2B3 in multiple cancer samples. All CC samples had SH2B3 expression < 0.1 in FPKM. Downregulation of this gene was also shown in CT1.

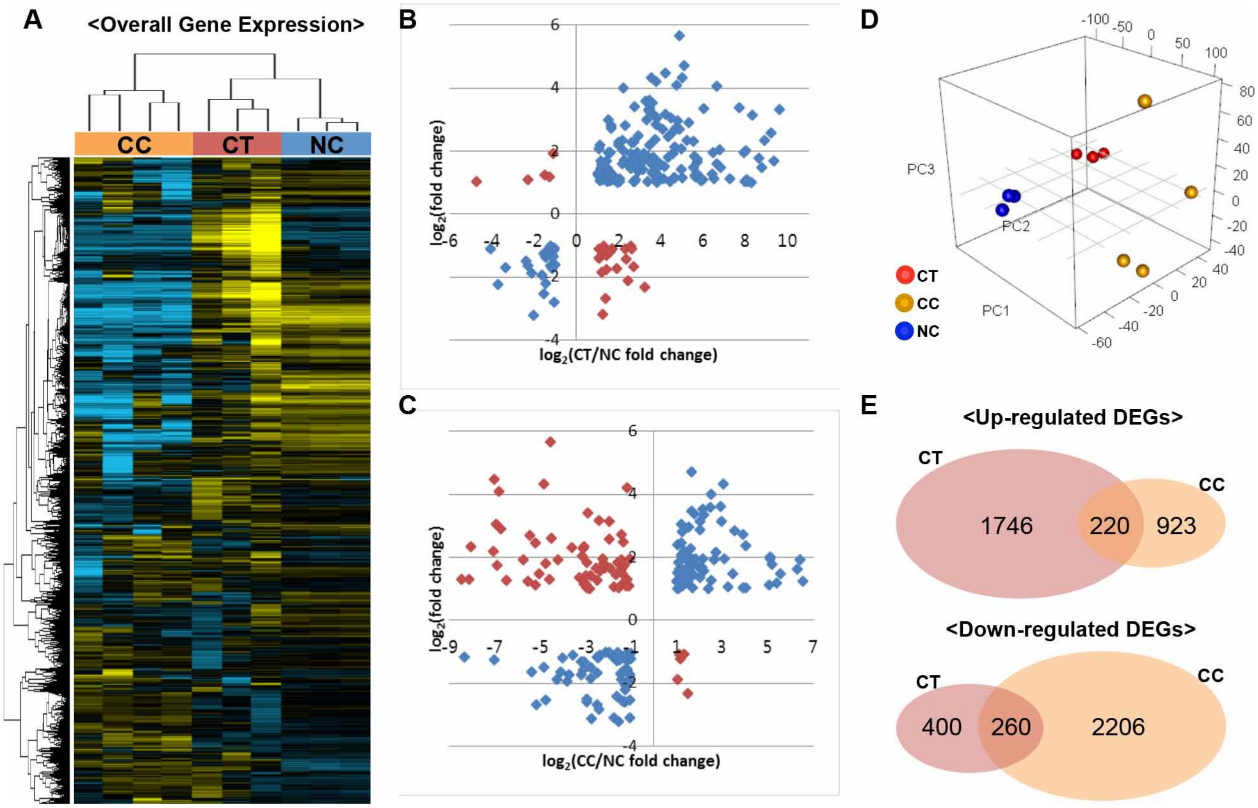

Supplementary Figure 4: Gene expression profiles of cancer tissues and cancer cell line samples. A. Hierarchical clustering resulted in a good categorization of study samples according to their cellular identity. CT, cancer tissue; CC, cancer cell line; and NC, normal cell line. **B.** The expression levels of DEGs reported by a previous study were consistent with those of CT samples. **C.** However, CC samples differed largely regarding upregulated genes, among which approximately half were downregulated in CC. Here, blue squares represent genes showing concordant expression patterns between the two, while red ones indicate discordant gene expressions. **D.** Principal component analysis separating study samples. PC2 differentiated cancer samples from normal samples. **E.** Comparison of CT and CC samples regarding DEGs. There were some shared components in gene expression between CT and CC. DEG, differentially expressed gene.

Supplementary Table 1: Whole exome sequencing and targeted sequencing summary

Supplementary Table 2: RNA-Seq summary

Supplementary Table 3: Mutation candidates from all flatforms (whole exome sequencing, targeted sequencing and RNA-Seq) (NS, nonsynonymous SNV; STOP, stopgain SNV; fsDEL, frameshift deletion; fsINS, frameshift insertion; nonfsDEL, nonframeshift deletion; nonfsINS, nonframeshift insertion)

Supplementary Table 4: Sanger sequencing result of *JAK3* hotspots

Supplementary Table 5: Fusion gene candidates

Supplementary Table 6: Differentially expressed genes from the DESeq2 analysis (*q*-value < 0.05)

Supplementary Table 7: Gene ontologies enriched with the DEGs common or different in CT and CC samples

Supplementary Table 8: Study subjects for sequencing and their clinical information (ANKL, aggressive NK-cell leukemia; ENKL, extranodal NK-cell lymphoma)

Supplementary Table 9: Candidates of targeted sequencing
